# High speed rail and coastal tourism: Identifying passenger profiles and travel behaviour

**DOI:** 10.1371/journal.pone.0179682

**Published:** 2017-06-23

**Authors:** Aaron Gutiérrez, Armando Ortuño

**Affiliations:** 1 Department of Geography, Rovira i Virgili University, Vila-seca, Tarragona, Spain; 2 Department of Building and Urbanism, University of Alicante, Alicante, Spain; Beihang University, CHINA

## Abstract

In this paper, we characterise tourists most likely to visit a coastal destination by high-speed rail (HSR). Our data came from a survey conducted among HSR passengers during 2014’s high season (July and August) at Spain’s Camp de Tarragona and Alicante Stations, each of which is near a mass tourism destination on the Mediterranean coast: the Costa Daurada and the Costa Blanca, respectively. We used responses to the survey, which presented binary discrete-choice situations, to construct a database necessary for a logistic regression model that allowed us to examine how passenger profile, trip characteristics, and stay conditions influenced the use of HSR services on visits to each coastal destination. Results highlighted significant differences in the profiles of tourists who arrived at each destination by HSR and, in turn, that no specific tourist profile is associated with HSR, even for two stations that serve sunny beach destinations. Among its implications, to analyse travellers that HSR can attract, it is vital to consider the specific characteristics of each destination and its current market.

## Introduction

In recent decades, as part of what some authors have called the ‘second railway age’ [1], European cities and provincial regions have experienced the expansion of the high-speed rail (HSR) network. Furthermore, the national agendas of many EU countries include plans to expand that network from its current 8,000 km of HSR track to 21,000 km by 2025 (according to data from the UIC, December 2015).

In that context, Spain’s HSR network has experienced the most significant growth in the last two decades, which has resulted in the country’s current 3,100-km network and 31 stations. Spain’s massive investment in HSR infrastructure, with more than €54 billion spent during this period, has prompted the consolidation of its HSR network into the world’s second largest, after China’s. Even despite the impact of the recent global financial crisis on Spain’s economy and public spending, the country continues to have the most ambitious plans in the EU for expanding its national HSR network in the coming years, with 1,909 km of HSR track currently under construction, according to December 2015 data from the Spanish Ministry of Public Works and Transport.

At the same time, though Europe is and will continue to be the world’s most important tourist destination, within which Spain’s Mediterranean coastal areas rank among the most vital tourist hotspots, relatively little applied research on what HSR implies for tourism has emerged. Nevertheless, among the complex combination of factors that contribute to tourism development, transportation has become critical [2]. In addition to travel time, accessibility, connectivity, and affordability, other factors inform travellers’ perceptions of destination attractiveness, including safety, comfort, and quality [3], as well as the quality of transportation infrastructure and accessibility, both of which have become key variables in developing tourist destinations and making them competitive [4]. As a result, a great deal of literature has analysed factors ranging from the contribution of investment in transportation infrastructure to improvements in the attractiveness of different destinations [5]. Although most of that research has focused on air instead of rail transportation [6–8], since HSR services can clearly affect tourist mobility, destination attractiveness, and transportation accessibility, the relationship between HSR and the tourism sector warrants as much attention as possible.

In this paper, we thus provide evidence to inform more thorough understandings of the profiles and travel behaviour of tourists who arrive at their destinations by HSR. More specifically, we examine how different explanatory variables influence the use of HSR services for travel to coastal destinations. To that end, we conducted a survey of HSR passengers at two Spanish HSR stations—namely, Camp de Tarragona and Alicante Stations—located near two Mediterranean mass tourism destinations: the Costa Daurada and the Costa Blanca, respectively. With the findings of those surveys, we constructed a logit model to identify the types of tourist most likely to visit each destination by HSR, all toward profiling tourists more likely attracted to mass tourism coastal destinations due to the existence of HSR services there.

We have organised our paper as follows. After this introduction, in Section 2 we situate our research among studies on the relationships between tourism and HSR. In Section 3, we characterise the two study areas, describe our data collection, and explain our methods, the results of which we present in Section 4, along with a discussion of major findings. We close the paper in Section 5 by highlighting our conclusions.

## Literature on HSR and tourism

Among the various recent studies that have identified the growing diversification of passengers who use HSR services [9–11], most have highlighted the significant role of tourism and leisure as reasons for using HSR services [12,13]. Although they have also recognised HSR’s increasing regional attractiveness, HSR’s real impact at destinations, particularly in terms of increasing number of visitors, remains controversial. Furthermore, although most of those studies agree that HSR can generate considerable opportunities for tourism development [14–17], some have not reported any significant impact whatsoever [18]. First studies on relations between HSR network expansion and regional development in Europe have questioned its contribution in tourism (see: Bonnafous [19] and Plassard [20] in France, Van den Berg and Pol [21] and Vickerman et al. [1] for the whole Europe, or more recently, Ureña et al. [11] and Garmendia et al. [22] for the Spanish context). At the same time, a growing body of literature has investigated competition between HSR and air transportation [23], and the substitution effects have been documented well [24–26]. Some studies have even highlighted the possible complementarity of both modes of transport [27,28], although the impact of such interactions on the tourism sector have yet to be studied in detail. One reason for that oversight could be that the question is especially sensitive, for air transportation remains the predominant mode of international tourism.

Unsurprisingly, most case study analyses on the relationship of HSR and tourism have been performed in Europe and focused on urban or business tourism, if not both. Such is the case with Bazin et al. [29], Coronado et al. [30], Guirao and Soler [12], Fachinetti et al. [31] and Ureña et al. [11], who studied different mid-sized cities in Spain and France, as well as with Pagliara et al. [32] and Delaplace et al. [33], who developed studies in metropolitan cities such as Madrid, Paris, and Rome. However, studies on the relationships of HSR and other types of tourism, including mass tourism destinations in coastal and mountain areas, remain few and far between.

Studies evaluating the effects of HSR on tourism have tended to adopt one of two general approaches. The first consists of different ex-ante methods for forecasting induced impacts based on core–periphery models, as used by Masson and Petiot [15] to investigate the Perpignan–Barcelona HSR line; multicriteria models, as used by Guirao and Campa [34] to examine Spain’s entire HSR network; gravitational models, as used by Wang et al. [35]to study China’s entire HSR network; or customer perceptions, as used by Becket and George [36] to speculate on the network planned to serve the US Gulf Coast. By contrast, the second consists of different ex-post methods, used by Guirao and Soler [12] on the case of the Toledo–Madrid HSR line and by [29] on the Paris–Lyon line, both of which involved passenger surveys to determine the relative importance of tourist traffic and to define tourist profiles and travel behaviour. Along with those case studies, Chen and Hayes [37]used multivariate panel analysis to argue that during 1999–2010, Chinese provinces with HSR services received 20% more international tourists and 25% more revenue than those without the infrastructure. Conversely, Albalate and Fageda [18] used data regarding the evolution of overnight stays in Spanish cities served by HSR during 1998–2013 to show that the presence of HSR services did not actively promote tourism.

These ex-post studies based on empirical data have provided results that have largely been complementary, but which in some cases have tended to differ. This shows that this remains a rather controversial field and one in which a wide range of factors, especially related to the territorial context, need to be taken into account. The existing literature on HSR agrees that this mode of transport is more competitive for medium-distance trips. Ureña et al. [11] have highlighted how, in territories with consolidated HSR networks, it has been possible to observe travellers changing from private cars to HSR for short-medium distance trips (between 100 and 400 km) and from air transport to HSR for medium-long-distance trips (between 400 and 700 km). In fact, recent studies have highlighted that one of the main effects of the recent extension of the HSR network in Europe has been to capture passengers from airlines [23,25]. This suggests that in most cases the main effects of HSR have tended to lie in the change in the mode of transport rather than in creating a greater degree of mobility. This implies that the capacity to attract new travellers to a specific destination as a result of the introduction of new HSR services could be less than expected [18]. However, the influence of HSR on destination choice is still an undeveloped field of study. In particular, there is a lack of empirical ex-post evaluation of the capacity of HSR to attract visitors to tourist destinations. Moreover, some studies stress that it is not possible to generalise about tourism development patterns associated with the extension of HSR networks [38–40]. Other researches highlight the positive effects of HSR on the tourism attractiveness of some destinations, especially when these are larger urban areas [32]. The improvement in accessibility [1], the reduction in travel time [41,42] and even—in some cases—the reduction in the cost of travelling have all helped to enlarge the potential market available to every city connected to the HSR network [11,35].

Taking that general context into account, we sought to contribute to literature on the relationship of HSR and tourism. A key source of added value in this paper is our use of two coastal destinations as case studies. That measure is a significant novelty, for all other studies in the field have focused on cities in which business tourism plays the starring role.

## Materials and methods

### Study areas: Alicante on the Costa Blanca and Tarragona on the Costa Daurada

**Fig 1:**
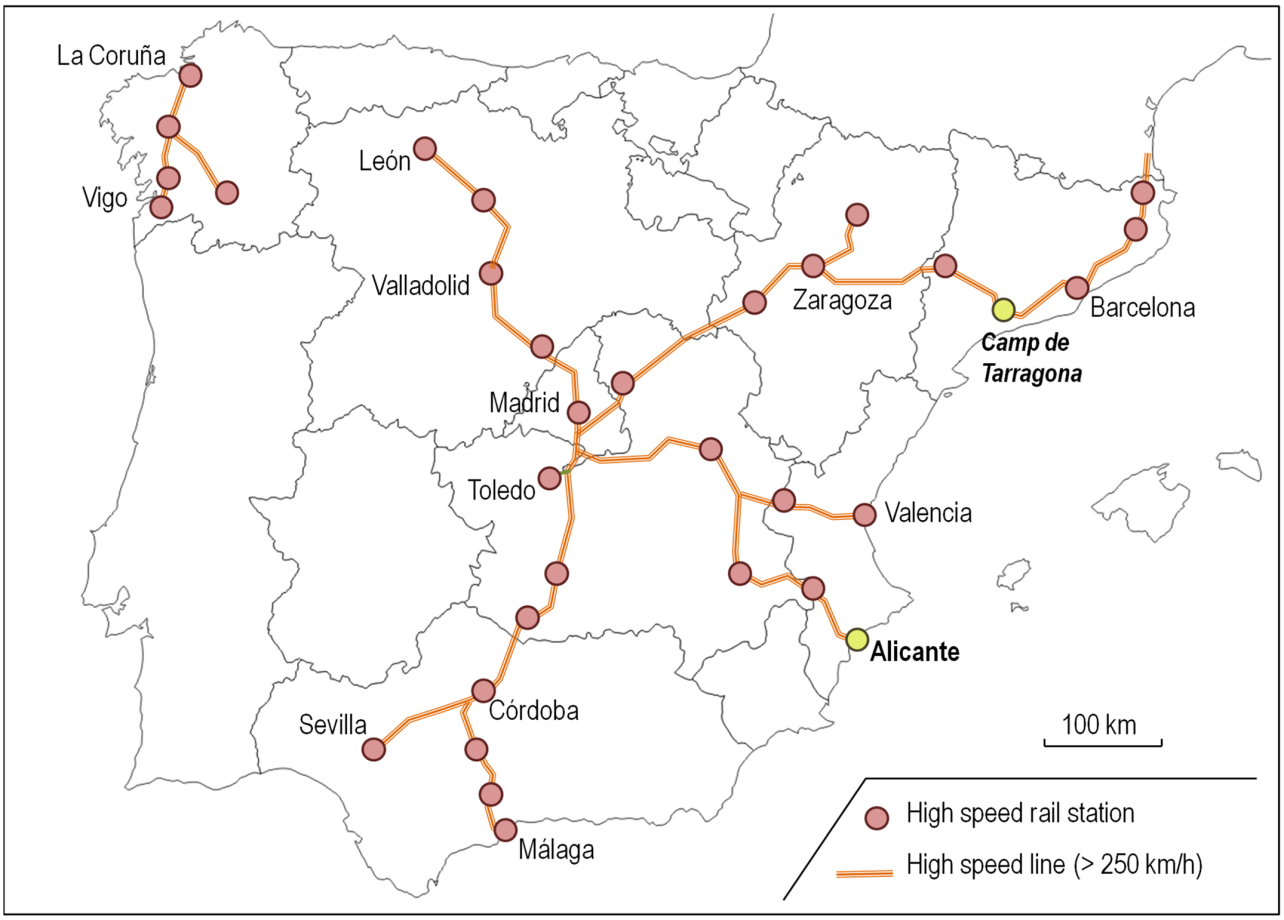
Spain’s HSR network.

We selected Alicante and Camp de Tarragona HSR Stations as case studies (Fig 1) because each is located near a coastal destination for mass tourism: respectively, the Costa Blanca and the Costa Daurada. As two of Spain’s most important coastal destinations, the Costa Blanca received more than 3.4 million tourists in 2013 and the Costa Daurada 2.7 million. Although Spain’s HSR network includes another station (i.e., Málaga) close to a coastal destination (i.e., Costa del Sol), Alicante and Camp de Tarragona present the most similar territorial contexts and destination characteristics.

On the one hand, the terminal Alicante Station has offered HSR services since June 2013 on the 550-km Madrid–Alicante HSR line, covered by nine trains per day in each direction in 135 min. In 2014, the station received 4.2 million passengers: 1.5 million by HSR and 2.7 million by other rail services. On the other, Camp de Tarragona Station is an intermediate node on the Madrid–Barcelona–French border HSR line, the primary corridor within Spain’s HSR network, that has received HSR services since December 2006. In February 2008, the station was connected to Barcelona and, in 2013, to Perpignan. The station currently receives 11 trains per day from Madrid (550 km, 150 min) and 22 from Barcelona (100 km, 40 min), each with the same number of return services. In 2014, it received more than 730,000 passengers, all on HSR trains, for the station does not offer other conventional railway services.

**Fig 2:**
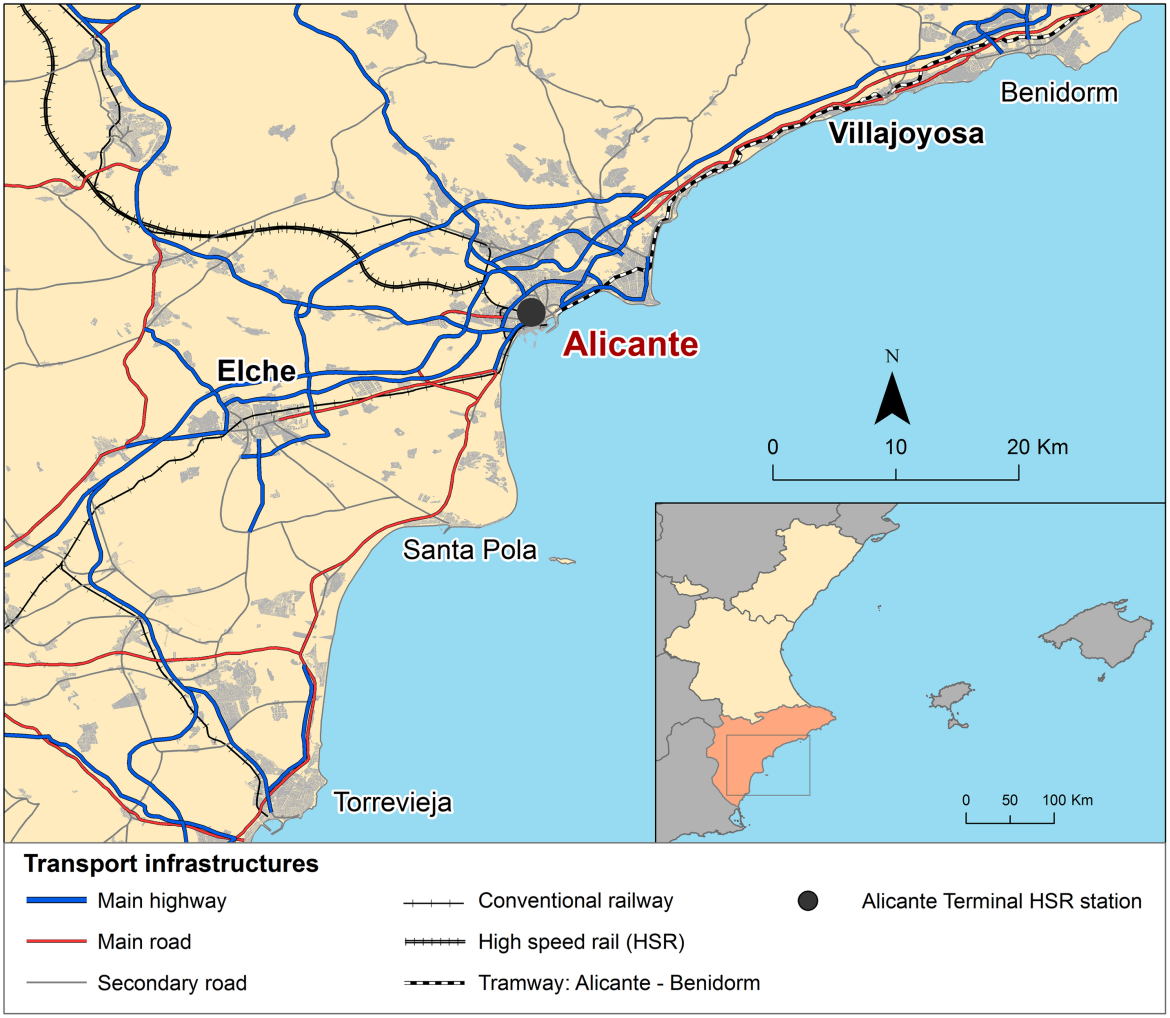
Alicante HSR Station and its regional context.

The attractiveness of both tourist destinations stems from their warm climates, the quality of their beaches, and the variety of leisure and cultural facilities in the area. The Costa Blanca boasts more than 25 km of beaches, punctuated by Benidorm and Torrevieja, with respective populations of 73,000 and 105,000 as of 2011, as the chief tourist centres. The area has a capacity for 70,000 overnight stays (Fig 2). By some contrast, the Costa Daurada has roughly 14 km of beaches, among which Salou and Cambrils, with respective populations of 27,000 and 34,000, as the most well-known destinations. It offers more than 120,000 beds for tourists, with an array of types of accommodation (e.g., campsites, hotels, and registered tourist apartments). Table 1 summarises the primary characteristics of tourists who arrived at both destinations in 2014, in data disaggregated by national and foreign tourists. In both places, Spaniards accounted for nearly 60% of total tourists received; most of them arrived by car and stayed at principally in second homes (Costa Blanca) and hotels (Costa Daurada). Conversely, foreign tourists arrived mostly by plane and stayed for longer periods.

**Table 1:** Tourist profiles in Alicante and Tarragona provinces, 2014.

	Alicante		Tarragona	
**Nationality**	Spanish (59.5%)	Foreign (40.5%)	Spanish (57%)	Foreign (43%)
**Specific origins**	Valencian Community (48.5%)	UK (47.4%)	Catalonia (45.5%)	France (28.2%)
	Community of Madrid (22.5%)	France (10.2%)	Aragón (15.0%)	Russia (25.7%)
		Germany (8.6%)	Basque Country (10.3%)	UK (18.9%)
**Accommodations (%)**	*Alicante—Spaniards*	*Alicante—foreign*	*Tarragona—Spaniards*	*Tarragona—foreign*
Hotel	21.6	31.2	41.1	66.2
Second residence	30.9	33.7	36.9	8.6
Friends or family second residence	34.6	27.6	9.6	4.3
Rented apartment	7.0	n.d.	8.9	14.2
Other	5.9	n.d.	3.4	6.6
**Mean length of stay**	5.8 nights	11.1 nights	7.8 nights	11.9 nights
**Transport mode (%)**	*Alicante—spaniards*	*Alicante—foreign*	*Tarragona—spaniards*	*Tarragona—foreign*
Car	88.6	10.5	81.6	31.3
Bus	5.9	n.d.	5.4	3.9
Train	3.5	n.d.	6.9	0.1
Plane	2.0	87.9	4.6	61.4
Other	0.0	n.d.	1.5	3.3

n.d.: No data. Source: Valencian Tourism Agency and Costa Daurada Tourism Observatory

**Fig 3:**
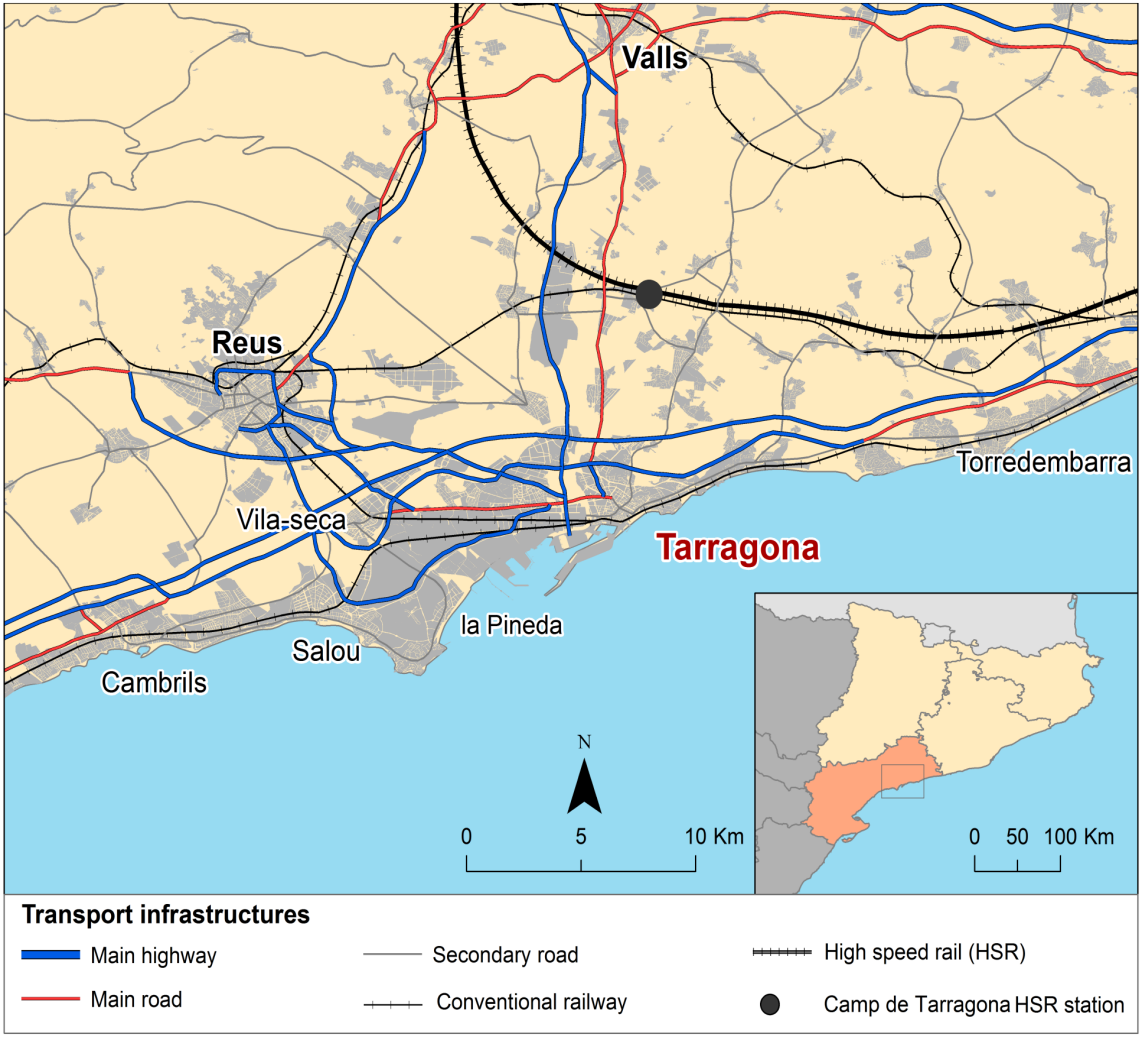
Camp de Tarragona HSR Station and its regional context.

Along with their central roles in tourism, the urban areas of both regions exhibit bicephalous structures. Tarragona, with 130,000 inhabitants, and Reus, with 105,000, are the two major demographic and economic poles in Tarragona Province (Fig 3), whereas Alicante, with 335,000 inhabitants, and Elche, with 228,000, play similar roles in Alicante Province. Both urban regions are also characterised by a sprawling, decentralised distribution of the population and activities, which has resulted in polycentric territorial structures [43].

The locations of the HSR stations within their respective regional contexts mark the most significant difference between the case studies. Alicante Station is located in the city centre of Alicante, and its HSR services are in a former conventional railway station now revamped to meet HSR requirements. Such a location facilitates intermodality, and the station is connected to the region’s major cities via both conventional rail and tram services. By contrast, Camp de Tarragona Station was built in 2006 entirely to receive HSR services. It is located peripherally at 14 and 17 km, respectively, from the cities of Tarragona and Reus and approximately 20 km from the chief tourist destinations on the coast. Its major deficiency is poor accessibility due to weak connectivity with the regional public transportation network [44,45]. Such differences in station location and their uneven accessibility from surrounding areas provide another interesting aspect for comparative analysis.

### Data and model specification

Our study focused on travellers returning from spending their summer holidays on the Costa Blanca in Alicante Province and the Costa Daurada in Tarragona Province. We collected data from surveys conducted with HSR passengers at both stations during the high season in July and August 2014. As coastal destinations, both stations received the greatest volume of tourists in summer. Previous surveys in both cases shown that the number of passengers for tourism and leisure motivations clearly decreased in other seasons [46,47]. A total of 423 passengers responded to the survey at Alicante Station and 574 at Camp de Tarragona Station, and we defined the sample size to achieve a 95% confidence level and 5% margin of error. Ultimately, we used a sample of 187 respondents for Alicante Station and 273 for Camp de Tarragona Station. All tourists surveyed were adults (>18 years old), gave their verbal informed consent, agreed to participate in the study, and were informed that the data would be analysed anonymously. The verbal consent was given individually previously to initiate each interview, and after being informed by the interviewers about the objective of the study and the subsequent treatment of the information obtained via the forthcoming interview. The verbal consent was the first question of the recorded interview and if it was not obtained the questionnaire is finished and it was not included in the study.

On both weekdays and at weekends, passengers who had holidayed at either destination and were waiting for HSR trains inside the station building were surveyed. The responsible committees of Spain’s public companies for train and railway infrastructures—Renfe and Adif, respectively—approved and supported the fieldwork and survey. The survey included questions intended to provide different types of information about the passengers, including their socioeconomic profile, region of residence, primary reason for travelling, type of train used, group size of the travelling party, mode of transport used from the station to their final destination, and other holiday characteristics (e.g., location, type of accommodation, and length of stay). The full questionnaire could be found in the Supporting Information (S1 File).

Results allowed us to construct tourist profiles via a logit model applied to several binary variables in order to produce a logistic regression. A great deal of literature on logistic regression has emerged since 1970, and it quite common to find recent research on discrete data analysis. Logistic regression models have been used in research on tourist demand [48], some using logit functions and codified survey results representing HSR passengers with binary variables [32]. Since the survey conducted at both stations involved a questionnaire with binary discrete-choice situations, we could use the responses to build the database necessary to devise a logistic regression model. We defined a logit regression equation as the inverse logistic regression equation, *P*_i_(*x*), shown below. We determined the coefficients of logistic equations *a* and *b*_k_ (*k* = 1, 2,…, *n*) by following a maximum likelihood approach that estimated the probability of the dependent variable *y* (i.e., a binary tourist variable), assuming a value of 1 for certain given values of predictor variables *x*_k_ (*k* = 1, 2,…, *n*).

$$P(y=1|\;x_{1,(\;X_{n}})=1/\left(1+\;e^{-(a+\Sigma_{n}b_{k}x_{k})}\right)$$

We could also express the logit regression equation as the inverse of the logistic function, *F* (*P*(*x*)):

$$F\left(P\left(x\right)\right)=\text{ln}\left(\frac{P_{i}\left(x\right)}{1-P_{i}\left(x\right)}\right)=\beta_{0}+\beta_{1}x_{1}+\beta_{2}x_{2}\ldots +\beta_{n}x_{n}$$

We therefore defined and applied the same logit models, with the same dependent and independent variables, to both cases in order to reveal the tourist passenger profile at each HSR station:

$$\text{Predicted logit }\left(\text{TOURIST}=1\right)=\beta_{0}+\beta_{1}\text{SEX}+\beta_{2}\text{AGE}+\beta_{3}\text{EDUC}\_1+\beta_{4}\text{EDUC}\_2+\beta_{5}\text{GROUP}+\beta_{6}\text{FAMILY}+\beta_{7}\text{DESTINATION}+\beta_{8}\text{DEPARTURE}+\beta_{9}\text{ACCOMODATION}+\beta_{10}\text{STAY}$$

in which the predicted (dependent) variable refers to the reason for travelling: TOURIST = 1 and 0 otherwise.

Explanatory variables related to sociocultural, travel, and stay characteristics and took into account several different independent variables with the aim of obtaining a model with high explanatory power to obtain the best possible profile of tourists arriving at each station. We defined explanatory variables included in the model based on information obtained via the survey. For variables with multiple-choice options (e.g., passenger origin and party structure), we selected options with more significant weight in at least one case, which contributed to achieving the greatest statistical significance and explanatory power for the model. We included the following explanatory variables:

SEX: 1 if the passenger was male; 0 if female.

AGE: 1 if the passenger was more than 41 years old; 0 if otherwise.

EDUC_1: 1 if the passenger had a secondary school or university education; 0 if otherwise.

EDUC_2: 1 if the passenger had a university education; 0 if otherwise.

GROUP: 1 if the passenger was travelling alone or in a couple; 0 if otherwise.

FAMILY: 1 if the passenger was travelling with his or her family; 0 if otherwise.

DESTINATION: 1 if the passenger travelled from the HSR station to the destination by public transport; 0 if otherwise.

DEPARTURE: 1 if the passenger had come from Madrid; 0 if otherwise.

ACCOMMODATION: 1 if the passenger had stayed in a hotel, holiday apartment, at a campsite, or in similar accommodation; 0 if otherwise.

STAY: 1 if the passenger had stayed at the destination for fewer than 8 nights; 0 if otherwise.

The full data with all the responses to the questionnaire in each station could be found in the Supporting Information (S2 and S3 Files).

## Results

### Descriptive statistics: Profiles of tourists who arrived at the destination by HSR

We aggregated data collected via the survey at each HSR station in three groups of items: trip characteristics (Table 2), passenger profile, and holiday characteristics (Table 2). The first item in Table 3 (i.e., Chief reason for travelling) related to the whole sample, whereas all other items in the table and in the following tables related exclusively to passengers whose chief reasons for travelling were tourism and leisure.

**Table 2:** Trip characteristics.

**Chief reason for travelling**	**Camp de Tarragona (%)**	**Alicante (%)**
**Tourism and leisure (at the destination)**	64.5	76.6
**Visiting family or friends**	9.3	13.2
**Shopping**	0.2	0.0
**Professional services (e.g., medical services)**	0.6	0.0
**Study**	0.3	1.9
**Work (i.e., commuting)**	3.1	0.0
**Professional or business**	20.7	6.0
**Other**	1.3	2.3
**Train typology**	**Camp de Tarragona (%)**	**Alicante (%)**
**AVE**	72.1	78.5
**Alvia**	22.0	21.5
**Other**	5.9	0.0
**Travel class**	**Camp de Tarragona (%)**	**Alicante (%)**
**Tourist class**	89.4	91.3
**First class**	9.2	8.7
**Unknown or no answer**	1.4	0.0
**Group size**	**Camp de Tarragona (%)**	**Alicante (%)**
**One**	50.0	23.4
**Two**	22.8	29.8
**Three**	12.6	13.2
**Four**	8.5	6.4
**More than four**	6.1	27.2
**Party structure**	**Camp de Tarragona (%)**	**Alicante (%)**
**Adult travelling alone**	50.0	23.4
**Family with children (<18 years old)**	20.2	24.5
**Adult relatives (≤35 years old)**	5.6	5.6
**Adult relatives (>35 years old)**	14.1	11.4
**Adult friends (≤35 years old)**	7.8	22.3
**Adult friends (>35 years old)**	2.3	6.0
**Group travel**	0.0	6.8
**Mode of transport from station to final destination**	**Camp de Tarragona (%)**	**Alicante (%)**
**Public transport (bus)**	15.1	15.1
Public transport (tram)	0.0	22.0
**Private car (i.e., friend or relative picked up the traveller from the station)**	61.9	14.7
Private car	0.0	6.0
**Taxi**	19.2	35.1
**Car rental**	1.2	1.1
**On foot**	0.0	4.5
**Other**	2.3	0.0

**Table 3:** Passenger profiles and tourist stay characteristics.

**Sex**	**Camp de Tarragona (%)**	**Alicante (%)**
Female	57.6	60.6
Male	42.4	39.4
**Age (in years)**		
18–25	15.4	29.1
26–40	38.3	26.8
41–60	34.8	21.2
>60	11.5	22.9
**Education level**		
None	1.3	3.4
Primary	13.1	10.8
Secondary	19.1	22.2
University	66.5	63.5
**Region of residence in Spain**	**Camp de Tarragona (%)**	
Catalonia	9.4	
Madrid	37.5	
Ebro River corridor	38.5	
Andalusia	6.8	
Elsewhere in Spain	7.8	
**Region of residence in Spain**	**Alicante (%)**	
Valencia	1.5	
Madrid	75.4	
Central Spain (excluding Madrid)	14.8	
Northern Spain	3.0	
Elsewhere in Spain	5.3	
**Destination**	**Camp de Tarragona (%)**	
Salou	25.5	
Cambrils	18.7	
Tarragona City	14.7	
La Pineda	6.6	
Elsewhere in Tarragona Province	34.5	
**Destination**	**Alicante (%)**	
Benidorm	37.4	
Alicante	35.1	
Torrevieja	5.9	
San Juan de Alicante	4.9	
Elsewhere in Alicante Province	16.7	
**Type of accommodation**	**Camp de Tarragona (%)**	**Alicante (%)**
Hotel	25.1	41.4
With friends or relatives	40.6	20.2
Second residence	19.5	18.7
Rented accommodation (e.g., apartment)	8.5	15.3
Other	5.3	4.4
**Length of stay**	**Camp de Tarragona (%)**	**Alicante (%)**
≤7 nights	70.7	63.5
>7 nights	29.3	36.5

For passengers at both stations, tourism and leisure were the top reasons for travelling, though that answer was more common at Alicante Station (76.6%) than at Camp de Tarragona (64.5%). This role of tourism and leisure as key travel motivation in summer at both stations is clearly related to their proximity to sun and beach destinations. As a result, other surveys to HSR passengers realised in both stations in winter denoted a decrease of this motivation for travelling: it supposed less than 16% in Camp de Tarragona [46] and 51% in Alicante [47]. By the other hand, during winter business motivated travel supposed more than 60% in the Camp de Tarragona and near to 30% in Alicante [46,47].

A key difference between the stations related to party structure. At Camp de Tarragona Station, 50% of the tourists were travelling alone, and 29.8% were travelling with a companion. As the number of members in their group increased, their weight within the whole sample decreased. By contrast, at Alicante Station most respondents were travelling with someone else (29.8%) or in a group of more than four people (27.2%); only 23.4% of respondents were travelling alone. The most common pattern at Alicante Station was travelling in a group of friends (28.3%) or in a family (25.5%). At Camp de Tarragona Station, those two groups accounted for 10.1% and 20.2%, respectively, of the total number of travellers surveyed.

Concerning mode of transport used to access the station, station location and the fact that Camp de Tarragona Station is a peripheral station and Alicante Station one in the city centre were notable. At Camp de Tarragona Station, 61.9% of tourists arrived by private car, whereas only 15.1% of tourists at Alicante HSR Station used that mode. Moreover, only 15.1% of tourists surveyed arrived to Camp de Tarragona station by public transport, while 37.1% used public transport in the case of Alicante. Within this context, the tramway from Benidorm to Alicante is the transport mode used by 22.0% of the tourists surveyed to reach the station.

The passenger profiles (Table 3) presented similarities in terms of reported education, which was significantly high in both cases.

The distribution of passengers by age differed notably by station. Nearly 23% of travellers at Alicante Station were more than 60 years old, as opposed to 11.5% at Camp de Tarragona Station. Alicante Station also had a greater presence of young people; 29.1% of its passengers were 25 years old or younger, as opposed to 15.4% at Camp de Tarragona Station.

Madrid was clearly the point of origin of tourists travelling at Alicante Station (75.4%). Although also a relatively important market for Camp de Tarragona Station (37.5%), slightly more tourists (38.5%) arrived there from destinations within the Ebro River corridor (e.g., the Basque Country, La Rioja, Navarra, and Aragon). Here it can be found a key difference between tourists arrived by means of HSR and general tourist in each destination (see Table 1). The medium-distance regions directly connected to Alicante and Tarragona by high speed line where those that concentrate the greatest volume of tourists. Madrid is the clearest example: it supposes the 22.5% and less than 10% of overall tourists arrived to Alicante and Tarragona (around 3.5 times less than HSR tourists in both cases). By the other hand, tourists from the same region (Community of Valencia and Catalonia) arrived through short-distance trips represents 48.5% and 45.5% of overall tourists, for 1.5% and 9.6% in the case of tourists arrived by means of HSR.

Spatial distribution patterns related to the final destination of tourists who arrived at each station by HSR also emerged. Tourists who arrived at Alicante Station tended to concentrate at Benidorm and Alicante city. By contrast, tourists at Camp de Tarragona Station used the station as a base for reaching a wider range of final destinations.

Type of accommodation revealed another key difference between the tourist destinations. Second residences represented the primary type of accommodation used by tourists at Camp de Tarragona Station (60.1%), whereas only 38.9% of tourists at Alicante Station used that type of accommodation. By contrast, hotels were the first option for tourists at Alicante Station (41.4%), as opposed to 25.1% of those at Camp de Tarragona Station. In the case of Camp de Tarragona, the type of accommodation of tourists arrived by means of HSR differs from overall tourists (see Table 1). The hotel is the main option, both for Spaniards (41.1%) and foreigners (66.2%). By the other hand, in the case of Alicante, the second homes become the main accommodation option for overall tourists (65.5% for Spaniards and 61.3% for foreigners). There are two factors that could explain these differences. Firstly, Alicante province has experienced during last decades a progressive specialisation in residential tourism. This issue explain the higher presence of second homes in the general tourist profile of Alicante [49,50]. Secondly, the HSR services in Camp de Tarragona station started in 2006, for 2013 in Alicante. Different studies had underlined that it is needed some years after the start of a new HSR service to experience significant transfers from travellers from other modes [11,22,51]. The tourists hosted in second home are those that have best knowledge of the destinations and usually repeat their visit various times during year. Their higher presence in HSR profile than the overall tourist profile in Camp de Tarragona, could then been explained because repeat tourists with greater knowledge of the transport options have changed their previous mode to HSR, as noted by [13].

### Model results: Probability of visiting the Costa Blanca and the Costa Daurada

The estimation results generated by the model appear in Table 4. The model for Camp de Tarragona presents a high pseudo *R*^2^ = 0.549, with five of the 11 factors proving significant. The variable related to age was significantly negative, meaning that it was highly probable that the tourist was less than 41 years old. Both variables related to level of education were significant, albeit with different signs; the first variable was positive, whereas the second was negative. That result meant that it was more likely that the tourist had only a secondary education. The variable GROUP was statistically significant with a positive sign, thereby implying that the tourist tended to travel with alone or with a companion. Lastly, results of the model highlighted a higher probability of the tourist’s staying on the Costa Daurada for fewer than 8 days.

**Table 4:** Logit estimation results.

	Alicante		Camp de Tarragona	
	Coefficient	St. Error	Coefficient	St. Error
SEX	0.1520	(0.7237)	-0.3269	(0.7873)
AGE	-1.2228	(0.7082)*	-1.9137	(0.7505)**
EDUC_1	2.3161	(1.1709)**	4.0013	(1.6474)**
EDUC_2	0.0115	(0.7011)	-2.2221	(1.6373)
GROUP	-2.0705	(0.7721)***	2.3324	(0.7458)***
FAMILY	-0.2742	(0.8716)	-1.5817	(0.6819)**
DESTINATION	-0.7483	(0.8632)	0.8988	(0.8325)
DEPARTURE	1.7621	(0.8409)**	1.1646	(0.9094)
ACCOMMODATION	2.0640	(0.7486)***	-0.6784	(0.9463)
STAY	-2.2272	(1.0277)**	1.9436	(0.723)***
INTERCEPT	2.8396	(1.0633)***	-0.6565	(1.4876)

Robust standard errors within parenthesis;

*Significant at 10%;

**Significant at 5%;

***Significant at 1%.

The tourist profile for Camp de Tarragona HSR Station was therefore of a person less than 41 years old with secondary education who was travelling alone or with a companion and stayed in Tarragona for fewer than 8 days.

Conversely, results generated by the model for Alicante Station showed a pseudo *R*^2^ = 0.327, with six of 11 variables proving significant. The variable related to level of education was statistically significant with positive sign, which pointed to the typical tourist’s being more likely to have completed secondary or university studies. The variable related to group size was significant with a negative sign, thereby implying a higher probability of tourists travelling in groups of three or more members. Different variables related to tourist characteristics were also significant and included the variable related to the point of origin (i.e., DEPARTURE), which was significantly positive, thereby indicating a greater probability of the tourist’s coming from Madrid than from other points of origin. Significant positive results for the ACCOMMODATION variable showed a greater probability of the tourist’s staying in holiday accommodation (e.g., hotel, campsite, or tourist apartments). The model also showed a greater probability of the tourist’s staying at the destination for at least 8 d, as highlighted by the STAY variable’s significantly negative sign.

The model therefore indicated that the average tourist arriving at Alicante HSR Station had a secondary or university education level, was travelling in group of three or more members, had arrived from Madrid, and stayed for at least 8 days.

Finally, Table 5 shows the average marginal effects of the model. They show that the education level, the size of the group and the length of the stay were the variables with greater incidence in the probability of the whole sample for both cases. These results present concordance with those obtained with the logit estimation model.

**Table 5:** Average marginal effects.

	Alicante		Camp de Tarragona	
	Coefficient	St. Error	Coefficient	St. Error
SEX	0.0079	(0.0376)	-0.0118	(0.0286)
AGE	-0.0633	(0.0399)	-0.0692	(0.0289)**
EDUC_1	0.1199	(0.0614)*	0.1447	(0.0586)**
EDUC_2	0.0006	(0.0363)	-0.0804	(0.0603)
GROUP	-0.1072	(0.0452)**	0.0843	(0.0307)***
FAMILY	-0.0142	(0.0444)	-0.0572	(0.0228)**
DESTINATION	-0.0387	(0.0467)	0.0325	(0.0324)
DEPARTURE	0.0912	(0.0443)**	0.0421	(0.0339)
ACCOMMODATION	0.1068	(0.0372)***	-0.0245	(0.0348)
STAY	-0.1153	(0.0553)**	0.0703	(0.0308)**

Marginal effects are probability changes because explanatory variables are discrete. Robust standard errors appear within parentheses.

*Significant at 10%;

**Significant at 5%;

***Significant at 1%.

## Conclusions

The estimated results of the model highlighted significant differences in the profile of tourists that are most likely to visit Costa Blanca or the Costa Daurada by HSR. As previous studies on HSR contribution to regional development highlighted, the impact of this infrastructure differs between cases, according to the different local development policies and territorial context [11,21,22,51]. Our study adds new evidences in the specific field of tourism development in coastal destinations.

The characteristics of the two regional contexts studied help to explain these differences. Tourists use the Camp de Tarragona Station to reach numerous scattered coastal settlements, whereas they use Alicante Station to reach the city of Alicante or Benidorm. The peripherally located Camp de Tarragona Station offers relatively poor public transport services, which requires a greater use of private cars to reach the final destination. Furthermore, the preferred accommodations of tourists that are most likely to visit Costa Daurada and Costa Blanca by HSR are second homes and hotel, respectively. That is the opposite of the general tourist profile in each case. It shows that tourists arrived by HSR have a specific and differentiated profile from overall tourists at destinations, and this profile differs among each case. Thus, tourists that are most likely to visit Costa Blanca by HSR tend to make longer stays, arrive in larger groups, and be older than those that are most likely to visit Costa Daurada. Consequently, our study reveals that there is not a unique profile for passengers using HSR services for tourism, even in the case of two stations that serve sun and beach destinations. It therefore follows that, to analyse travellers who might use HSR services, it is crucial to consider the specific characteristics of each destination and its current market. Moreover, the findings of the study allow us to state that there is not a predefined target market for HSR services for tourist purposes.

## Supporting information

S1 File Questionnaire for HSR passengers at Alicante and Camp de Tarragona stations. (PDF)

S2 File Alicante responses data. (XLSX)

S3 File Camp de Tarragona responses data. (XLSX)
