# Supplementary material for: High speed rail and coastal tourism: Identifying passenger profiles and travel behaviour
Source: PLoS One. 2017 Jun 23;12(6):e0179682. doi: 10.1371/journal.pone.0179682 (PMC5482456; doi:10.1371/journal.pone.0179682)
Supplement: S1 File — (PDF) [file pone.0179682.s001.pdf]

ENGLISH

P0 is the filter question: Our study is focused on tourist comming back to their origin after spent their holidays in Costa Daurada or Costa Blanca

**0. What is the main reason of your journey?**

I take the train every day to go to work (commuting) – end of the questionnaire

It is a business / professional trip – end of the questionnaire

Family reasons / to visit my family - end of the questionnaire

I take the train to attend go to the University /high school (for studies matters) – end of the questionnaire

To attend professionals services (i.e. visiting a doctor) – end of the questionnaire

Shopping – end of the questionnaire

It is for tourism / holidays / leisure - end of the questionnaire

I am returning back to my home (not Tarragona/Alicante) after spent my holidays in Costa Daurada / Costa Blanca – end of the questionnaire

Other reason – end of the questionnaire

In this case, note the reason: _______________________

**1. What railway services are you going to use?**

AVE

AVANT

INTERCITY

TRENHOTEL

Unknown/No answer

**2. What is the travel class of your ticket?**

Tourist class

First class

Unknown/No answer

**3. You arrive to this area by means of this railway?**

Si – Go to question 4

No – Go to question 3b

**3b- What transport mode did you use to reach the destination?**

- By train (using another railway station in the province).

- By plane

- By private car

- By other mode of transport

**4. What is the destination (station) of your current trip? (Returning from holidays)**

---

**5. Where is your main residence?**

In case of Spaniards – please, provide the province

In case of foreigners – please, note the country

**6. What is the size of the group that you are travelling with? (number of members)**

__________________________________________________________

**7. How had the trip being organized?**

I booked a package holiday in a travel agency

I booked the trip directly

**8. In what kind of accommodation have you stayed?**

Hotel

Staying in friends or relatives second residence

Second residence

Rented accommodation (apartment)

Other

**9. What transport mode did you use for the transfer from station to final your destination?**

Public transport (bus)

Public transport (tram)

Private car (someone collecting the traveller from the station)

Taxi

Car rental

On foot

Private car

Other

**10. What was the main destination (municipality) of your holidays?**

_______________________________________

**11. Is it your firs visit to Costa Daurada/Costa Blanca?**

Yes – Go to question13

No – Go to question 12

**12. If no, Did you use the station in past visits?**

Yes – Go to question13

No – Go to question 14

**13. If yes, how many times?**

____________________________________________________________________

**14. What is the lenght of your stay at Costa Daurada/ Costa Blanca?**

More than 7 nights

7 nights or less

**15. Who had you come with for these holidays?**

Adult travelling alone

Family with children (<18)

Adult relatives (≤35)

Adult relatives (>35)

Adult friends (≤35)

Adult friends (>35)

Group travel

**16. Gender**

Man

Woman

**17. Age**

18-25 years

26-40 years

41-60 years

> 60 years

**18. What is your highest study level reached?**

No studies

Primary (Basic/elemental school level )

Secondary (high school level)

University

(Graduate / postgraduate / doctorate)

At the end of the interview it is noted the date and hour in which each questionnaire has being done.
